# Enhancement of immune cytokines and splenic CD4+ T cells by electroacupuncture at ST36 acupoint of SD rats

**DOI:** 10.1371/journal.pone.0175568

**Published:** 2017-04-13

**Authors:** Longyun Chen, Anli Xu, Nina Yin, Min Zhao, Zhigang Wang, Tao Chen, Yisheng Gao, Zebin Chen

**Affiliations:** 1 Department of Biochemistry, Basic Medical College, Hubei University of Chinese Medicine, Wuhan, China; 2 Department of Anatomy, Basic Medical College, Hubei University of Chinese Medicine, Wuhan, China; 3 Department of Pathogenic Biology, Basic Medical College, Hubei University of Chinese Medicine, Wuhan, China; University of North Dakota School of Medicine and Health Sciences, UNITED STATES

## Abstract

Electroacupuncture at the ST36 acupoint can enhance the body’s immune function. However, the mechanism for this enhancement has not been fully described. Our study was designed to investigate the effect of electroacupuncture on the immune function of Sprague-Dawley (SD) rats. The rats were randomly divided into three groups: a control group, a non-acupoint group (abdominal muscle acupuntured) and a ST36 acupoint group. Our results showed that successive electroacupuncture at the ST36 acupoint for 3 d significantly enhanced the interferon-γ (IFN-γ) level in the serum of SD rats. The results also showed that the serum and extracts from spleen cells of the ST36 acupoint group contained higher levels of interleukin (IL)-2 and IL-17 compared to those of the other two groups. Immunohistochemical analysis showed that electroacupuncture applied to the ST36 acupoint enhanced the expression level of CD4 in spleen cells. Furthermore, it was observed that CD4 co-localized with transient receptor potential vanilloid (TRPV) channels at the membrane of splenic CD4+ T cells and the expression level of CD4 was related to TRPV channels in the electroacupuncture treatment. These observations indicated that electroacupuncture stimulation at the ST36 acupoint enhanced the level of immune cytokines and splenic CD4+ T cells through TRPV channels in this system.

## Introduction

Electroacupuncture (EA) treatment is one of the most popular approaches in complementary medicine and health maintenance. It has been shown to have some analgesic effects in relieving pain [1–5]. Recently, some experimental and clinical studies have shown that sequential electroacupuncture stimulation applied to the ST36 acupoint (Zusanli acupuncture point) performed well in the treatment of stress-induced immunodeficiency [5, 6]. A previous study demonstrated that electrical stimulation at the ST36 acupoints can activate the immune system and help support anti-cancer treatments [7–9]. However, the curative mechanisms of electroacupuncture remain poorly understood, which limits the application of electroacupuncture on a larger scale.

CD4+ T cells are one of the important cells in the human immune system. T cells play a very important role in the immune system. CD4+ T cells, which are known as the "helper" of the immune system, protect the body against microbes such as viruses. The CD4+ T cell is an important component of the adaptive immune response. After different stimuli, CD4+ T cells can differentiate into different subsets of cells, including Th1 and Th2 cells, follicular helper (Tfh) cells, Th17 cells, and regulatory T cells (T_REG_). The functions of the CD4+ T cell are diverse, such as the activation of immune cells, direct cytotoxic effects, and immunoregulation [10].

Free Ca^2+^ ions are used as second messengers for most cells, including immune cells. Resting T cells and B cells are known to maintain low intracellular Ca^2+^ concentrations [11]. Some receptors located on the immune cell surface are known to increase the concentration of intracellular Ca^2+^, such as the T cell receptor, the B cell receptor and co-stimulatory receptors [12]. A previous study also noted that Ca^2+^ signaling in immune cells is important for the differentiation of immune cells and gene transcription [11–13]. It has been shown that the activation of T cells can cause an increase in the IFN-γ and IL-2 levels [14]. Further, electroacupuncture has been demonstrated to enhance the levels of splenic IFN-γ and IL-2 released by helper T cells [15, 16]. How the increases in the IFN-γ and IL-2 levels were induced by electroacupuncture requires further study, and the effect of electroacupuncture on splenic T cells needs to be explored. Hence, the aims of this study were to investigate the effect and mechanism of electroacupuncture on the release of immune cytokines and the activation of splenic T cells.

## Materials and methods

### Animals and electroacupuncture treatment

**Fig 1:**
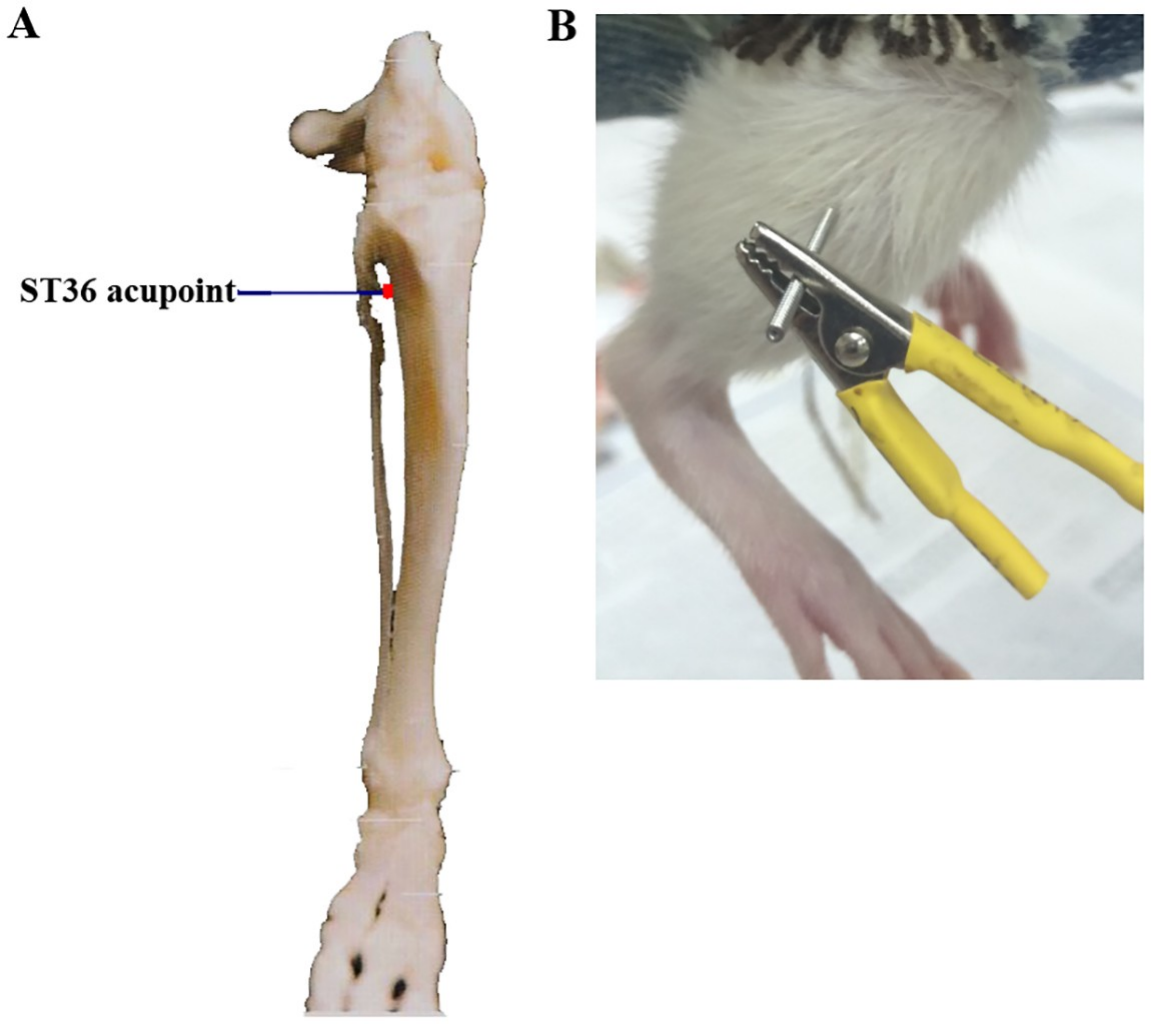
(A and B) The location of ST36 acupoint on the body of SD rats.

Adult male Sprague-Dawley (SD) rats weighting 200±25 g were purchased from the Experimental Animal Center of Hubei University of Traditional Chinese Medicine (medical laboratory animal certificate number: SCXK (Hubei) 2015–0018). TRPV1 knockout (TRPV1 -/-) mice were purchased from Nanjing Biomedical Research Institute of Nanjing University (medical laboratory animal certificate number: SCXK (Jiangsu) 2015–0001). All the animals were kept in the Experimental Animal Center of Hubei University of Chinese Medicine. The Experimental Animal Center of Hubei University of Chinese Medicine obtained a laboratory animal administrative license from the Science and Technology Department of Hubei Province (number: SYXK (Hubei) 2012–0067). The animals were randomly divided into 3 groups: (a) a control group in which the animals received no treatment and were housed at the animal facilities (20–23°C, 50% ± 5% humidity, 12-hour light/dark cycle with lights on at 8:00 am); (b) a non-acupoint group in which electroacupuncture stimulation was applied to the abdominal muscle as described in a previous study [16]; (c) and a ST36 acupoint group in which electroacupuncture stimulation was applied to the ST36 acupoint. The location of ST36 acupoint was shown in Fig 1A and 1B. Two sterilized acupuncture needles (diameter 0.16 mm, length 25 mm, Zhongyantaihe, Tianjin, China) were inserted at the ST36 acupoint or the external oblique abdominal muscle to a depth of 4–5 mm, and the needles were connected by their handles to a HANS (Han’s Acupoint Nerve Stimulator) electro-stimulator. The disperse-dense waves at the alternating frequencies 2 Hz and 15 Hz and an intensity of 1 mA were used as electrical stimulation for 30 min. The stimulation protocol was repeated each day for 1, 3, 7 or 14 days. Female animals were excluded from our study. All manipulations were performed between 8:00 a.m. and 12:00 a.m. every day to minimize the influence of circadian rhythms. After the electroacupuncture treatment, the animals were anesthetized by an intraperitoneal injection with 50 mg/kg pentobarbital sodium for the following experiments. All experiments were performed at room temperature. All the animals were allowed to calm down in the new environment for at least 30 min.

### Preparation of extract from ST36 acupoint area

To prepare the extract, the rats were anesthetized by an intraperitoneal injection with 50 mg/kg pentobarbital sodium. Then, the tissues around the ST36 acupoint (10 mm length, 10 mm width and 5 mm depth) were cut off, weighed, minced into small pieces and homogenized in cold PBS (10 mg tissue per 100 μl PBS). The resulting suspension was subjected to further break down via ultrasonication, and the homogenates were then centrifuged at 1500 g for 15 min. The supernatants were collected and stored at -80°C.

### ELISA assay

An ELISA assay was used to assess the IFN-γ, IL-2 and IL-17 levels in the serum and extracts from the ST36 acupoint area. After the electroacupuncture treatment, one eye of the anesthetized rats was pulled out using tweezers, and the fresh blood was collected in a 10 ml EP tube. The tubes were maintained at 4°C for 24 h, and the serum was harvested and stored at -80°C. The serum and extracts were subjected to an enzyme-linked immunosorbent assay (ELISA) to detect IFN-γ, IL-2 and IL-17 expression according to the manufacturer’s protocol (Thermo Fischer). All assays were done at least three times in three separate experiments.

### Immunohistochemical analysis

The tissues were dehydrated using sequential incubations with 75%, 85%, 90%, 95% and 100% ethanol. The tissues were treated with a transparent agent after the dehydration. Transparent tissues were soaked and embedded in paraffin wax. They were sliced and placed on slides. The slides were immersed in xylene for 10 minutes. Then, the sections were dehydrated by sequential incubations with 100%, 95%, 80% and 60% ethanol for 5 minutes each. The sections were rinsed with distilled water three times for 3 minutes each. The slides were transferred to a microwave-proof container and covered with citrate buffer for antigen retrieval. Sections were blocked using 5% normal blocking serum for 1 hour. Then, they were incubated with the respective primary antibody for 1 hour at room temperature. Following primary antibody incubation, the slides were incubated in a sufficient amount of peroxidase-labeled polymer for 30 minutes and the substrate for 5–10 minutes, successively, until a brown color developed. Then, the slides were treated by hematoxylin counterstaining. After dehydration and mounting, the slides were observed under a microscope.

### SDS-PAGE and WB

The tissues of the spleen were cut out, weighed and minced into small pieces as before. Then, the tissues were homogenized in lysis buffer (20 mM Tris-HCl [pH 8.0], 100 mM NaCl, 1.9% [wt/vol] Triton X-100, 1 mM 1,4-dithiothreitol, and 5% [vol/vol] glycerol) on ice for 30 min. The homogenates were centrifuged at 12,000 rpm for 5 min at 4°C. After centrifugation, the supernatants were collected. The concentration of the protein in each sample was determined using the Bradford method. Equal amounts of protein were separated by 12% SDS-PAGE and transferred onto a nitrocellulose membrane (GE Healthcare). The membrane was blocked with 5% nonfat milk and incubated with the primary antibodies for IL-2 and IL-17, followed by incubation with the goat anti-rabbit secondary antibodies.

### Detection of Ca^2+^ concentration

The serum was subjected to a colorimetric method for the detection of the Ca^2+^ concentration according to the manufacturer’s protocol (Calcium Assay Kit, BioVision).

To detect the Ca^2+^ concentration in spleen cells, the fresh spleen tissue was made into a single cell suspension (2 x 10^6^ cells per EP tube). The cells were resuspended in serum-free medium at a final concentration of 2.5 μmol/L of Fluo 3-Am. The suspension was placed at 37°C at 5% CO_2_ for 30 min. The suspension was centrifuged at 1500 rpm for 5min, and the supernatant was discarded. The cells were washed with calcium-free PBS twice and suspended in 500 μl calcium-free PBS. The Ca^2+^ concentration of the spleen cells was detected by flow cytometry.

### Immunofluorescence analysis

The spleen was treated using the method described in the immunohistochemical analysis with different primary and secondary antibodies. The specific primary Abs for CD4, TRPV1 or TRPV4 were used. Following the primary antibody incubation, the goat anti-rabbit IgG (Cy3) or goat anti-mouse IgG (FITC) fluorescence secondary antibodies were added. DAPI was used to stain the nucleus for 5 min.

## Results

### Effect of electroacupuncture on the expression of IFN-γ

**Fig 2:**
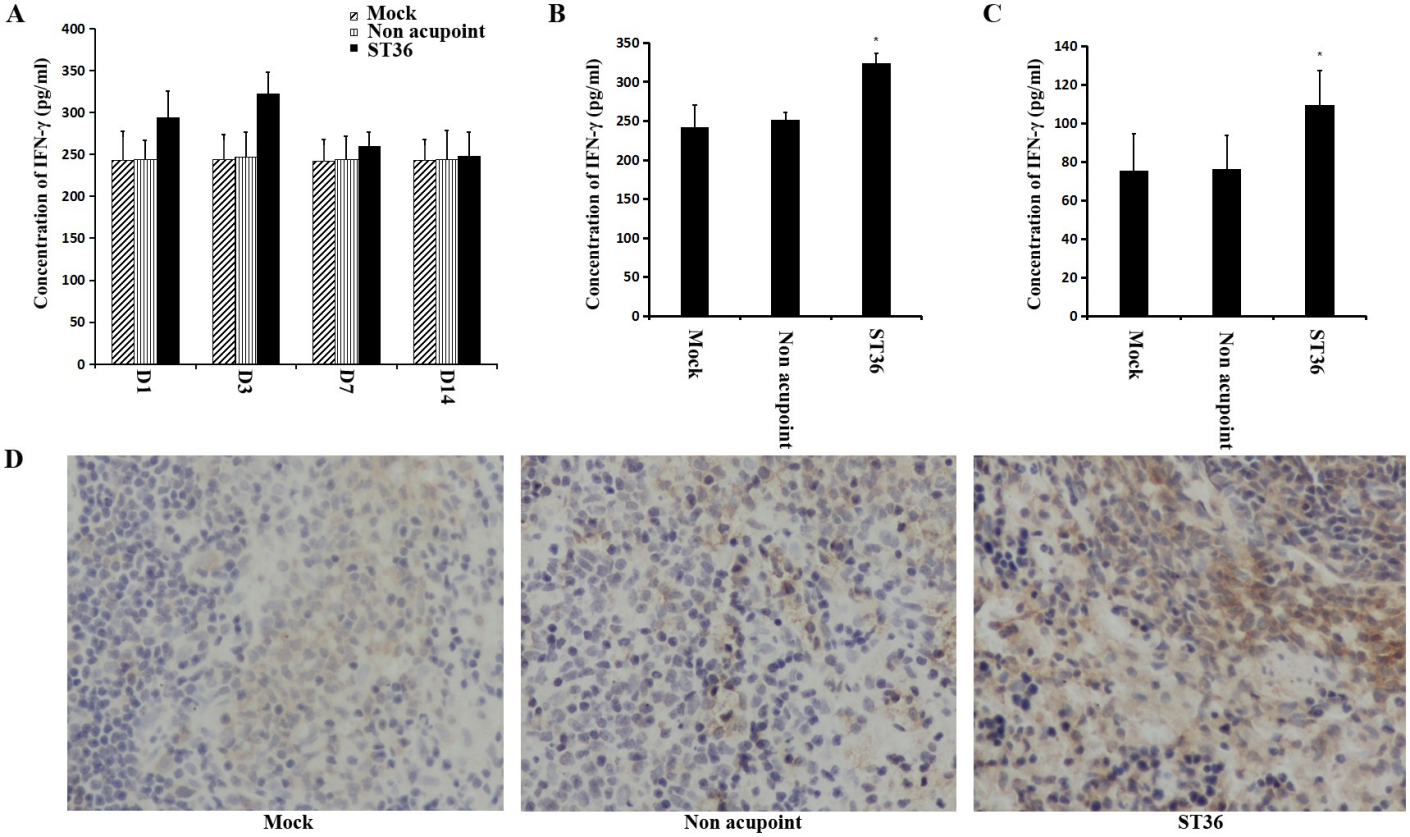
Effect of electroacupuncture at ST36 acupoint on IFN-γ level of SD rats. (A) Electroacupuncture was applied to the left and right ST36 acupoint in the same rat for 30 min. The electroacupunture stimulation was repeated each day for 1, 3, 7 or 14 consecutive days on different rats. The IFN-γ level in serum of SD rats was detected. (D1 = 1 day) (B) Electroacupuncture at the ST36 acupoint caused the IFN-γ level in serum increased. *P<0.05 for ST36 acupoint group versus control group and non acupoint group. (C) After the rats were treated by electroacupuncture at the ST36 acupoint, the IFN-γ level in extracts from ST36 acupoint area increased. *P<0.05 for ST36 acupoint group versus control group and non acupoint group.(D) The IFN-γ level in spleen was detected via immunohistochemical analysis. The positive signal is brown or dark brown. Mock = Control; Non acupoint = electroacupuncture at abdominal muscle; ST36 = electroacupuncture at the ST36 acupoint.

A previous study reported that electroacupuncture applied to the ST36 acupoint for three consecutive days enhanced the level of IFN-γ in the spleen of rats [15, 17]. Thus, we wanted to determine whether electroacupuncture causes the level of IFN-γ in the serum to increase. Male SD rats were divided into three groups: a control group, a non-acupoint group and a ST36 acupoint group. For the non-acupoint group, electroacupuncture was applied to the abdominal muscle. Electroacupuncture at the ST36 acupoint was applied to the SD rats for 1, 3, 7 or 14 consecutive days. The level of IFN-γ in the serum was detected via the IFN-γ ELISA assay. The results showed that electroacupuncture at the ST36 acupoint for three consecutive days significantly increased the IFN-γ level in serum (Fig 2A and 2B). Based on these results, the electroacupuncture treatment at ST36 acupoint was only applied for three consecutive days in our residual research. We further studied the effect of electroacupuncture on the IFN-γ level in the extracts from the ST36 acupoint area. The IFN-γ ELISA assay demonstrated that the IFN-γ level in the extracts of the ST36 acupoint group increased because of the electroacupuncture (Fig 2C). Consistent with a previous study [15], our results demonstrated that electroacupuncture at the ST36 acupoint enhanced the level of IFN-γ in the spleen, as shown by immunohistochemical analysis (Fig 2D).

### The effect of electroacupuncture applied to the ST36 acupoint on the IL-2 and IL-17 levels

**Fig 3:**
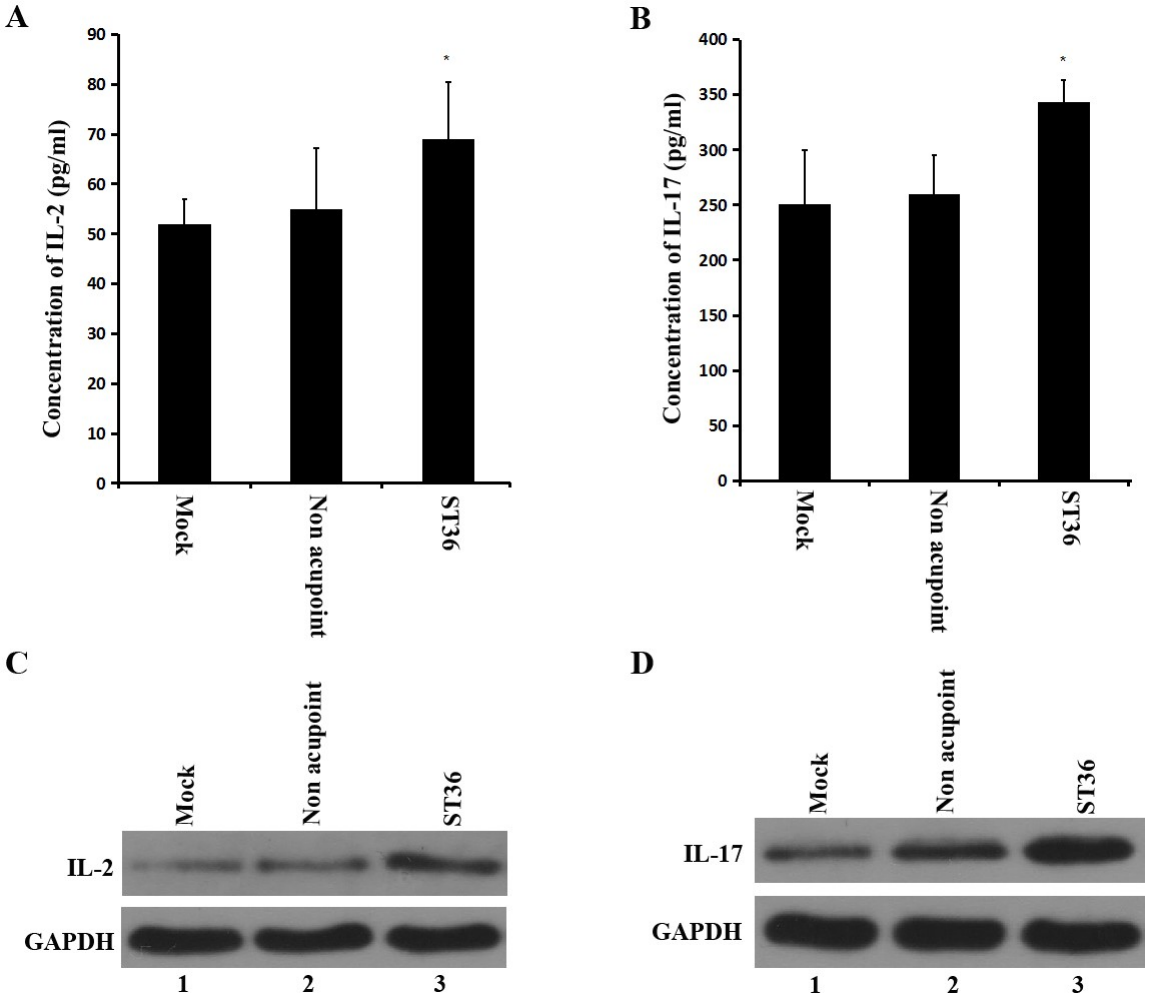
Effect of electroacupuncture at ST36 acupoint on IL-2 and IL-17 levels of SD rats. (A and B) IL-2 and IL-17 level of serum increased in rats treated by electroacupuncture at the ST36 acupoint. *P<0.05 for ST36 acupoint group versus control group and non acupoint group. (C and D) The expression levels of IL-2 and IL-17 in extracts from spleen cells were analyzed via western blot. GAPDH was used as control.

Because IFN-γ, IL-2 and IL-17 are the cytokines released by helper T cells, we examined whether electroacupuncture at the ST36 acupoint enhanced T-cell cytokine production. First, the serum and the tissue extract were prepared, and the expression level of the endogenous cytokines was examined. As shown in Fig 3A and 3B, the IL-2 and IL-17 levels in the serum of the ST36 acupoint group were significantly higher than those of the non-acupoint and control groups. However, there was no difference between the acupunctured abdominal muscle and those of the control rats.

To determine whether the IL-2 and IL-17 levels were also increased in the extracts from the spleen, western blot analysis was used. As shown in Fig 3C and 3D, the increases in the IL-2 and IL-17 levels were accompanied by electroacupuncture at the ST36 acupoint. These findings indicated that the electroacupuncture applied to the ST36 acupoint activated helper T cells and enhanced the release of T-cell cytokines.

### Electroacupuncture applied to the ST36 acupoint enhanced splenic CD4+ cells

**Fig 4:**
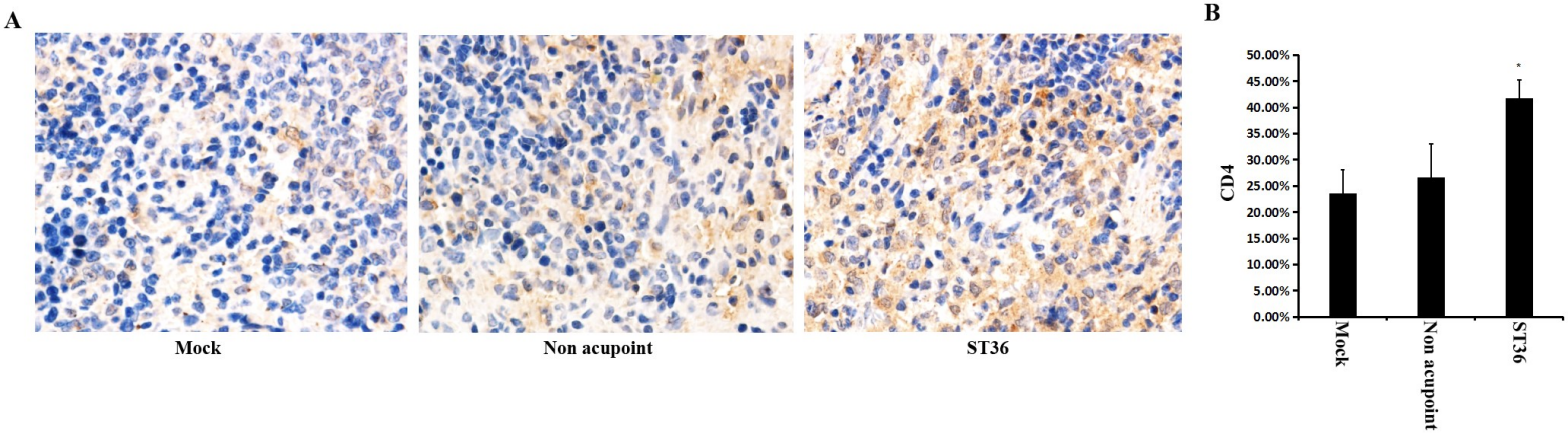
The expression level of CD4 in spleen was assayed via immunohistochemical analysis. The positive signal is brown or dark brown. (A) The expression level of CD4 increased in spleen cells of the rats treated by electroacupuncture at the ST36 acupoint. (B) Proportions of immunopositive CD4+ T cells.

It is known that CD4 molecules mainly distribute on the surface of T helper cell subsets. To investigate the effect of electroacupuncture on helper T cells, immunohistochemical analysis was used. Fig 4A and 4B show that the electroacupuncture applied to the ST36 acupoint significantly increased the proportion of T cells with the expression of CD4. This result may suggest that the enhancement of the differentiation and proliferation of splenic CD4+ T cells was induced by electroacupuncture stimulation at the ST36 acupoint.

### Effect of electroacupuncture on the distribution of Ca^2+^

**Fig 5:**
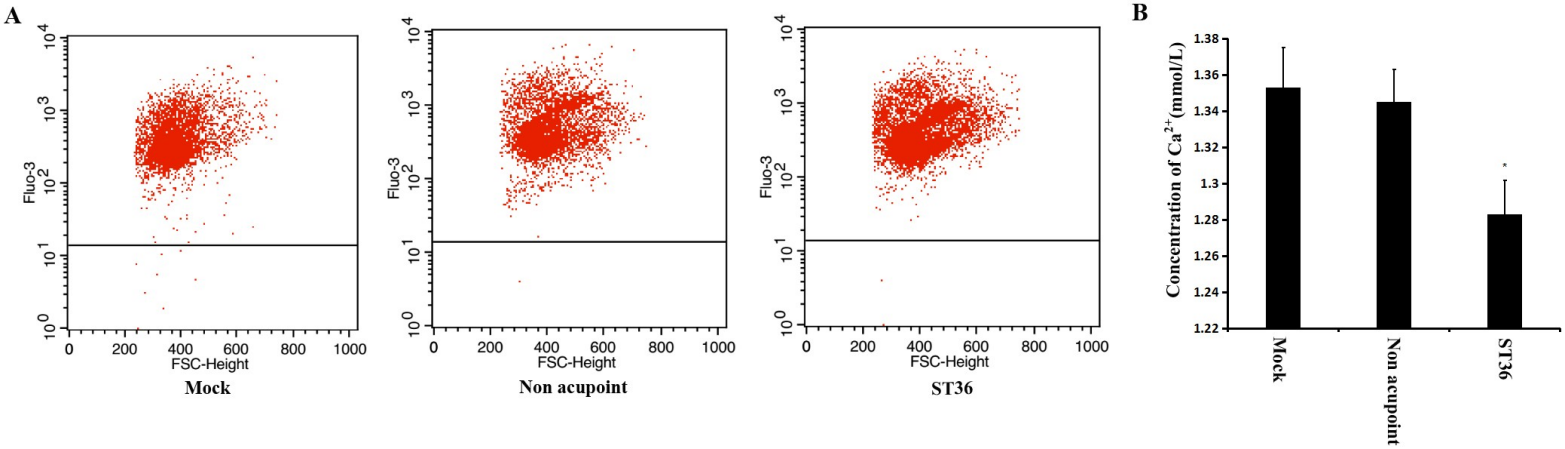
The concentration of Ca^2+^ in spleen cells and serum. (A) The values mentioned in the upper corner of each flow cytometric dot-plot indicate the concentration of Ca^2+^ in spleen cells. Representative dot-plots of three independent experiments are shown. The concentration of Ca^2+^ increased in spleen cells of the rats treated by electroacupuncture at the ST36 acupoint. (B) The concentration of Ca^2+^ in serum of the rats in each group was detected. Electroacupuncture at the ST36 acupoint caused the decreasing of Ca^2+^ content in serum. *P<0.05 for ST36 acupoint group versus control group and non acupoint group.

It has been reported that a higher level of intracellular Ca^2+^ is important for IL-2 receptor expression, IL-2 production and T cell activation [18]. We assessed whether the level of intracellular Ca^2+^ was changed by electroacupuncture. The concentration of Ca^2+^ in the spleen cells was detected via flow cytometry. As shown in Fig 5A, the intracellular Ca^2+^ level of the ST36 acupoint group was higher than that of the non-acupoint and control groups. Because the level of intracellular Ca^2+^ increased, we hypothesized that the level of extracellular Ca^2+^ may decrease. To check this, we detected the Ca^2+^ concentration in the serum by using a colorimetric method. The results showed that the Ca^2+^ concentration in the serum decreased because of the electroacupuncture at the ST36 acupoint (Fig 5B). These findings support the idea that electroacupuncture applied to the ST36 acupoint led to Ca^2+^ influx in spleen cells.

### TRPV1 and TRPV4 located at the surface of splenic CD4+ T cells

**Fig 6:**
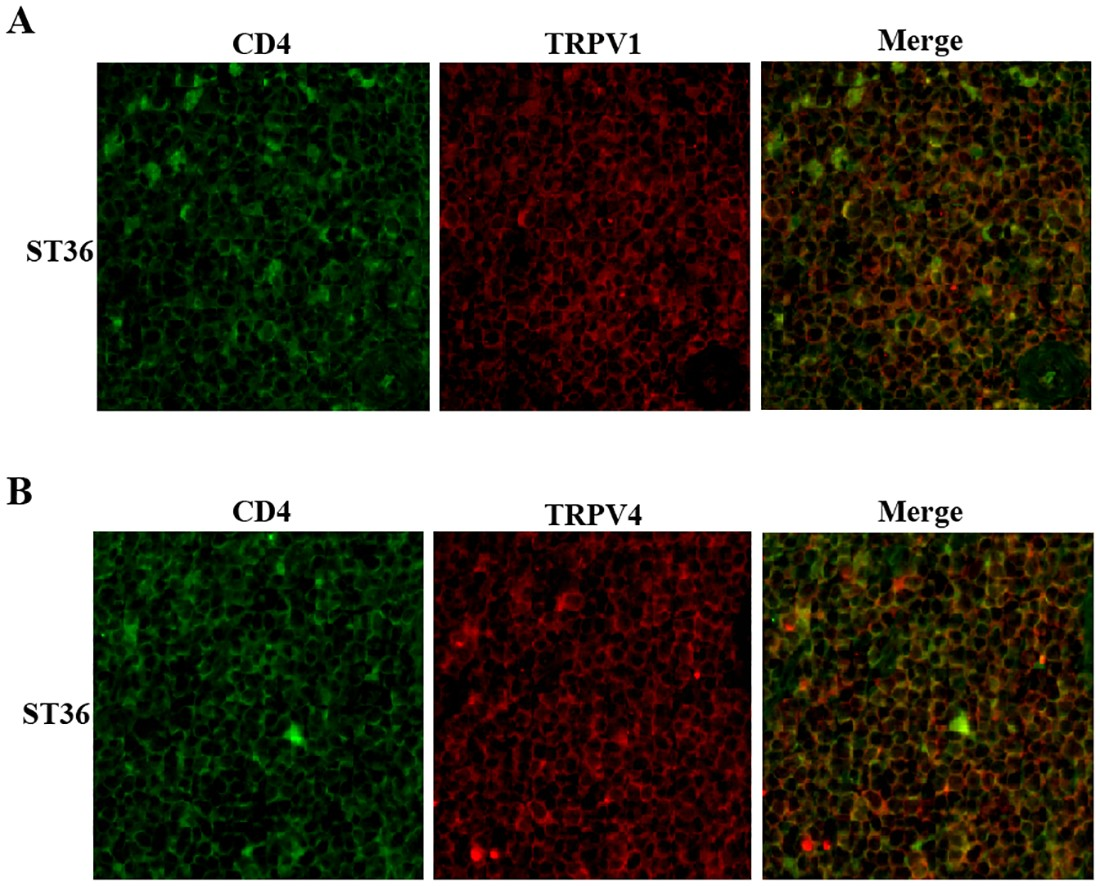
CD4 and TRPV channels co-localized at T cell membrane. (A) Confocal images demonstrated that CD4 and TRPV1 co-localized in T cell membrane. Cells were immunostained with specific antibodies for CD4 and TRPV1. (B) CD4 and TRPV4 co-localized at T cell membrane.

TRPV channels are a group of non-selective cation channels in T cells. A previous study demonstrated that TRPV1 and TRPV4 belong to the TRPV subfamily and are expressed endogenously in human and mice T cells. The activation of TRPV1 and TRPV4 leads to a Ca^2+^ influx in purified murine T cells [14]. It has also been reported that electroacupuncture at the ST36 acupoint has an effect on TRPV1 and TRPV4 [19, 20]. To probe the correlation between the CD4 and TRPV channels, we detected the expression of TRPV1 and TRPV4 in CD4+ T cells via immunofluorescence. We found that TRPV1 and TRPV4 co-localized with CD4 at the membrane of splenic CD4+ T cells (Fig 6A and 6B). This finding suggested that electroacupuncture at the ST36 acupoint led to a Ca^2+^ influx in splenic T cells via TRPV channels.

### Effct of electroacupuncture at ST36 acupoint on TRPV1 -/- mice

**Fig 7:**
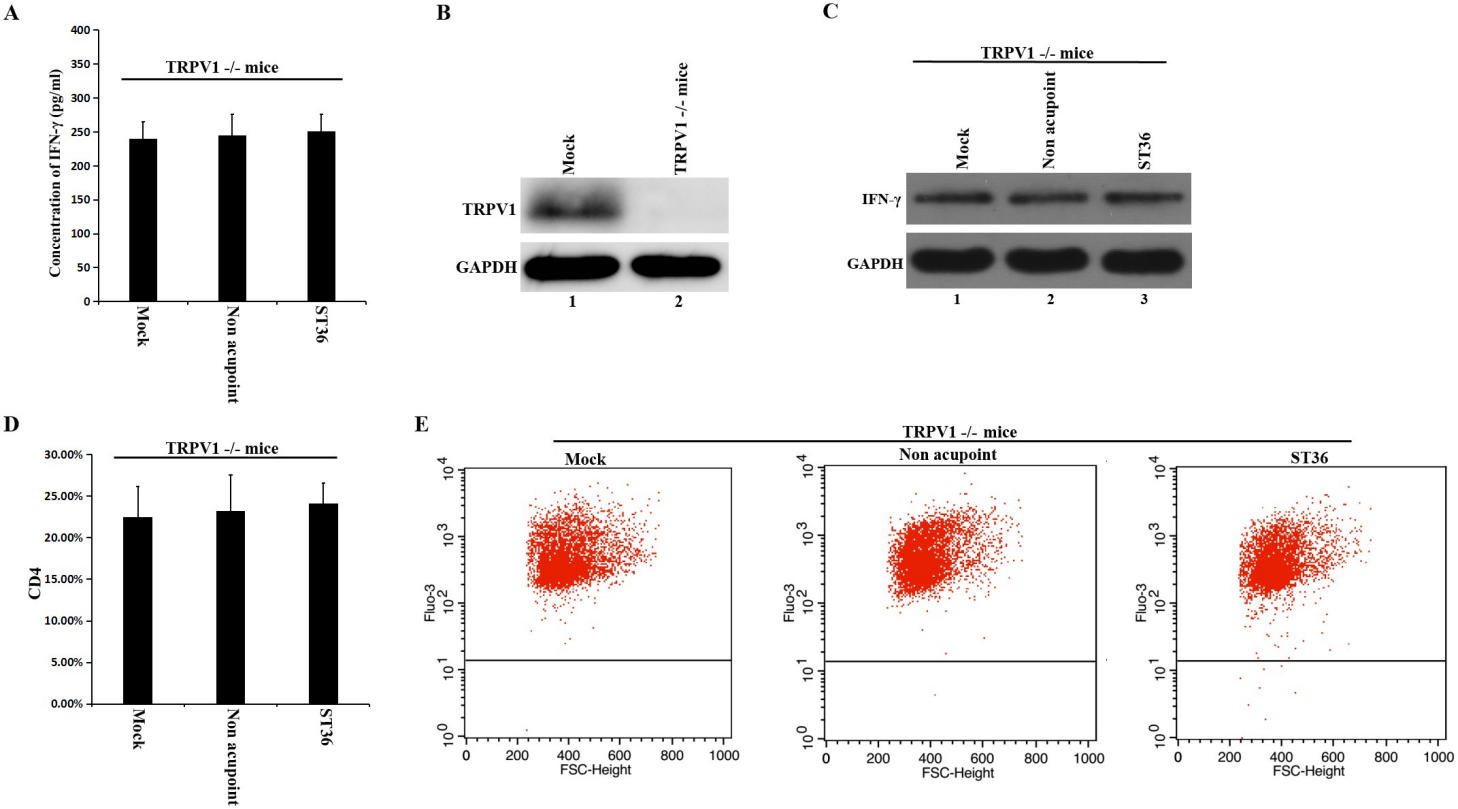
Electroacupuncture at ST36 acupoint had little effect on TRPV1 -/- mice. (A) The IFN-γ levels in serum of TRPV1-/- mice were detected. (B) The expression of TRPV1 in spleen was detected. (C) The expression levels of IFN-γ in spleen were detected via western blot. GAPDH was used as control. (D) Proportions of immunopositive CD4+ T cells in spleen of TRPV1 -/- mice. (E) The concentration of Ca^2+^ in spleen cells of TRPV1 -/- mice. The values mentioned in the upper corner of each flow cytometric dot-plot indicate the concentration of Ca^2+^ in spleen cells. Representative dot-plots of three independent experiments are shown. The concentration of Ca^2+^ had no significant increase in spleen cells of the TRPV1 -/- mice treated by electroacupuncture at the ST36 acupoint.

In order to check if the TRPV is necessary for electroacupuncture enhanced immune function, electroacupuncture at ST36 acupoint was applied to TRPV1 -/- mice. The results showed that the expression level of IFN-γ had no obvious change in serum and spleen of TRPV1 -/- mice with the electroacupuncture at ST36 acupoint (Fig 7A, 7B and 7C). Without TRPV1, there is no significant increase in the proportion of T cells with the expression of CD4 under electroacupuncture treatment (Fig 7D). We also found that the concentration of Ca^2+^ in the spleen cells of the ST36 acupoint group with TRPV1 knockout didn’t increase significantly (Fig 7E). These results suggested that electroacupuncture at ST36 acupoint had little effect on TRPV1 -/- mice.

## Discussion

In China, acupuncture, especially at the ST36 acupoint, has a long history of use as a supplementary therapy for the treatment of acute and chronic diseases. Although the detailed mechanisms underlying electroacupuncture need further study, several studies have indicated that local molecular and cellular changes occur at and around the location of the acupoint. Some mechanisms have been proposed to explain the effects of electroacupuncture, including 1) the extracellular signal-regulated kinase (ERK) pathway [20, 21], 2) the regulation of the nervous control system (neurotransmitter regulation) [22, 23], and 3) an increase in function of the immune system [8, 15, 16]. In addition, it has been suggested that the morphological changes induced by acupuncture in fibroblasts and connective tissue are involved in the therapeutic effect of acupuncture [24, 25]. However, the exact mechanisms by which acupuncture favorably increases the function of immune system are not well known. Our present study clearly showed that electroacupuncture stimulation at the bilateral ST36 acupoint once a day (30 min) for 3 days significantly enhanced the levels of IFN-γ, IL-2 and IL-17 (Figs 2 and 3). It also showed that electroacupuncture applied to the ST36 acupoint increased the number of CD4+ T cells in the spleen compared to those observed in the non-acupoint and control groups (Fig 4A and 4B). These results indicated a significantly positive correlation between the levels of immune cytokines and CD4+ T cells.

CD4 is a marker for helper T cells. IL-2 is primarily released by activated helper T cells [26]. In the immune system, IL-2 serves as an important factor in the up-regulation of natural killer (NK) activity. IFN-γ can be released by both helper T cells and IL-2-activated NK cells [27–29]. IL-17 is released by IL-17-producing CD4+ T cells (Th17 cells). As a growth factor for both effector and regulatory T cells, IL-2 has been shown to enhance the indirect relationship that exists between T_REG_ and Th17 cells. A previous study demonstrated that T_REG_ can be converted into IL-17-expressing CD4+ T cells in the presence of exogenous IL-2 [30]. Considering these reports and the present results, it is possible that the enhancement of IFN-γ, IL-2 and IL-17 by electroacupuncture at the ST36 acupoint is due, in part, to the differentiation and activation of CD4+ T cells.

It is generally accepted that Ca^2+^ signaling is important in the context of T cell activation and differentiation. The involvement of Ca^2+^ in the regulation of the immune system has been demonstrated via the modulation of extracellular and intracellular Ca^2+^ concentrations using several Ca^2+^ chelating agents. It has been reported that EGTA, as a chelator of extracellular Ca^2+^, inhibits the rise in cytoplasmic Ca^2+^, IL-2 production and the further proliferation of naive T cells in response to activation stimuli [31]. Additionally, T cell activation mediated by concanavalin A (ConA) was sustained for a longer time because of the immediate rise in intracellular Ca^2+^. Similarly, the expression of T cell antigen receptor β (TCR-β) is regulated by the depletion of intracellular Ca^2+^ [18]. Considering these reports, we assessed the level of intracellular Ca^2+^ in spleen cells. The results showed that the level of intracellular Ca^2+^ increased because of electroacupuncture at the ST36 acupoint (Fig 5A). At the same time, a decrease in the Ca^2+^ concentration in the serum was observed (Fig 5B). These results proved that the rise in the intracellular Ca^2+^ induced by electroacupuncture at the ST36 acupoint was responsible for the differentiation, proliferation and activation of splenic CD4+ T cells.

TRPV1 and TRPV4 are non-selective Ca^2+^ ion channels belonging to the TRPV subfamily. They are endogenously expressed and function in the splenic T cells of mice [14]. The functional role of TRPV1 and TRPV4 in the context of T cell activation and effector functions has been described in a previous study. In this study, it was found that Ca^2+^ influx was induced by TRPV1 and TRPV4 activation in purified murine T cells. If TRPV1 and TRPV4 were inhibited by specific inhibitors, the ConA-driven mitogenic activation of T cells decreased to significant levels. T cell activation was almost abolished by the combination of TRPV1 and TRPV4 inhibitors. This finding indicates that TRPV1 and TRPV4 play an important role in T cell activation. It also has been reported that the inhibition of these channels had a strong inhibitory effect on the release of cytokines, such as TNF, IL-2 and IFN-γ [14]. From Fig 6A and 6B, we can see TRPV1 and TRPV4 were expressed and co-localized with CD4 at the membrane of splenic T cells. In previous study, it has been reported that electroacupunture at ST36 acupoint had effect on TRPV1 [32]. So we checked the effect of electroacupuncture treatment on TRPV1 -/- mice. Our results showed that electroacupuncture at ST36 acupoint had little effect on TRPV1 -/- mice (Fig 7). According to these results, we propose that the activation of TRPV channels by electroacupuncture at the ST36 acupoint are responsible for the Ca^2+^ influx in spleen cells.

As a critical component of the adaptive immune system, T lymphocytes mediate protection against infection and malignancy but are also involved in many immune pathologies. T-cell development plays a key role in regulating adaptive immune responses. It is also used as a model system for studying cell differentiation. T-cell development has been of interest to immunologists for a long time. The effect of acupuncture on immune function and the treatment of acute and chronic diseases has been demonstrated by clinical and experiment research. Our findings indicated that electroacupuncture at the ST36 acupoint enhance immune function through the activation splenic T cells.

In conclusion, the present study has demonstrated that electroacupuncture at the ST36 acupoint was able to regulate the production of immune cytokines (IFN-γ, IL-2 and IL-17) and the differentiation and activation of splenic T cells, which was mediated by the regulation of extracellular and intracellular Ca^2+^ concentrations. Our results also suggested that Ca^2+^ influx in spleen cells induced by electroacupuncture at the ST36 acupoint might be mediated by TRPV channels. Further research on the mechanism of how electroacupuncture enhances immune function is currently being carried out.

## Supporting information

S1 Table Specific criteria for monitoring SD rats. We took notes on food intake, water intake and body weight of SD rats in a week to monitor animal health. Electroacupuncture treatment was applied from day 5 to day 7.
(XLSX)

S2 Table Specific criteria for monitoring TRPV1 knockout mice. We took notes on food intake, water intake and body weight of TRPV1 knockout mice in a week to monitor animal health. Electroacupuncture treatment was applied from day 5 to day 7.
(XLSX)

S1 File Additional details about the care and use of animals utilized in this research. (DOC)
