# Supplementary material for: Enhancement of immune cytokines and splenic CD4+ T cells by electroacupuncture at ST36 acupoint of SD rats
Source: PLoS One. 2017 Apr 13;12(4):e0175568. doi: 10.1371/journal.pone.0175568 (PMC5391063; doi:10.1371/journal.pone.0175568)
Supplement: S1 File — (DOC) [file pone.0175568.s003.doc]

**According to the Journal requirements, we uploaded additional details about the care and use of animals utilized in this research (Please see supporting information ).**

**1. We took notes on food intake, water intake and body weight of all the animals in a week to monitor animal health. Electroacupuncture treatment was applied from day 5 to day 7 (Please see the excel).**

**2. There were no animals became severely ill or died at any time prior to the experimental endpoint**

**3.We euthanized animals by anesthesia. All the animals were anesthetized by an intraperitoneal injection with 150 mg/kg pentobarbital sodium for euthanasia.**
